# Web Resources for SARS-CoV-2 Genomic Database, Annotation, Analysis and Variant Tracking

**DOI:** 10.3390/v15051158

**Published:** 2023-05-12

**Authors:** Yexiao Cheng, Chengyang Ji, Hang-Yu Zhou, Heng Zheng, Aiping Wu

**Affiliations:** 1 School of Life Science and Technology, China Pharmaceutical University, Nanjing 211100, China; 2 Institute of Systems Medicine, Chinese Academy of Medical Sciences & Peking Union Medical College, Beijing 100005, China; 3 Suzhou Institute of Systems Medicine, Suzhou 215123, China

**Keywords:** SARS-CoV-2, web resource, database, annotation, genomic analysis, variant tracking

## Abstract

The SARS-CoV-2 genomic data continue to grow, providing valuable information for researchers and public health officials. Genomic analysis of these data sheds light on the transmission and evolution of the virus. To aid in SARS-CoV-2 genomic analysis, many web resources have been developed to store, collate, analyze, and visualize the genomic data. This review summarizes web resources used for the SARS-CoV-2 genomic epidemiology, covering data management and sharing, genomic annotation, analysis, and variant tracking. The challenges and further expectations for these web resources are also discussed. Finally, we highlight the importance and need for continued development and improvement of related web resources to effectively track the spread and understand the evolution of the virus.

## 1. Introduction

As of February 2023, the pandemic of the coronavirus disease 2019 (COVID-19) has affected more than 750 million confirmed cases and more than 6 million deaths globally (https://covid19.who.int/, accessed on 5 February 2023), causing severe health and economic burden worldwide. The etiologic agent of COVID-19 is severe acute respiratory syndrome coronavirus 2 (SARS-CoV-2). SARS-CoV-2 is a single-stranded positive RNA virus with a genome of approximately 30,000 nucleotides in length. It is a member of the species *Severe acute respiratory syndrome-related coronavirus*, subgenus *Sarbecovirus*, genus *Betacoronavirus* [1,2]. Its genome contains four structural proteins (S, E, M, and N), eight accessory proteins, and sixteen nonstructural proteins [3].

Since the first sequence of SARS-CoV-2 was published [1], its sequences have been generated and shared in unprecedented numbers. As of February 2023, more than 10 million SARS-CoV-2 sequences have been deposited in public databases [4,5,6,7,8,9,10].

Genomic epidemiology has played an important role during the pandemic. In the early days of the pandemic, phylogenetic analysis revealed the early international spread of SARS-CoV-2 and highlighted the importance of public health measures in preventing onward transmission [11,12]. There have been many variants of SARS-CoV-2 that have emerged since its first detection, and some variants have spread rapidly in many countries or regions. Understanding such variants’ introduction and transmission dynamics is crucial for adjusting public health measures. Using phylogenetic and epidemiological approaches, researchers continued to monitor and track the transmission of SARS-CoV-2 variants during the pandemic [13,14,15,16,17,18,19,20]. Contact tracing and superspreading events were also investigated by combining genomic sequence and epidemiological evidence [21,22,23,24,25,26,27,28].

The genomic data have been extensively used to track the evolution of SARS-CoV-2. After the emergence of SARS-CoV-2, researchers conducted a phylogenetic analysis of more than one hundred genomes to preliminarily estimate the virus’s origin time and evolutionary rate [29]. As SARS-CoV-2 continues to mutate, genetic diversity of the virus is discovered both within and between individual hosts [30,31,32,33,34,35,36,37,38,39,40,41,42,43]. Several SARS-CoV-2 variant nomenclatures have been proposed, which have important implications for virus surveillance, functional analysis, and public communication [44,45,46]. To prioritize global monitoring and research, the World Health Organization (WHO) designated variants that pose an increased risk to global public health as variants of concern (VOCs) using letters of the Greek alphabet [46]. Some of the VOCs have many defining mutations and display a discontinuous pattern of evolution [47,48,49,50,51,52]. Many researchers speculated that such variants might come from patients with chronic infections [50,51,52,53,54,55,56,57,58,59,60,61,62], but there is no direct evidence of the origin of these variants. Viruses can evolve and adapt to their environments or hosts [63]. Although SARS-CoV-2 has only been circulating in the human population for a few years, signals of adaptive evolution have been detected [43,47,49,64,65,66,67,68,69].

Scientists used bioinformatics tools or web resources for the genomic analysis of SARS-CoV-2 [70,71,72]. The ongoing pandemic greatly impacted the development of bioinformatics tools or web resources, and lots of resources specific to SARS-CoV-2 were developed. Compared with a tool with a command-line interface or graphical user interface, a web resource is a straightforward way to analyze and display the SARS-CoV-2 genomic data. In the context of real-time generation and sharing of SARS-CoV-2 genomic data, real-time genomic analysis based on web resources allows us to better monitor and understand the virus.

**Figure 1:**
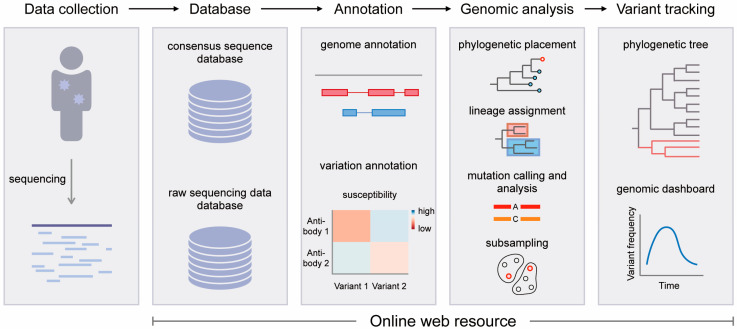
Schematic workflow for SARS-CoV-2 genomic research using web resources. The web resources for SARS-CoV-2 genomics can be divided into four categories: database, annotation, genomic analysis, and variant tracking.

This review covers current web resources related to SARS-CoV-2 genomics that are still being maintained or updated (Figure 1 and Table 1). These web resources can be divided into four categories according to their main functions: database, annotation, genomic analysis, and variant tracking.

## 2. SARS-CoV-2 Genomic Databases

**Figure 2:**
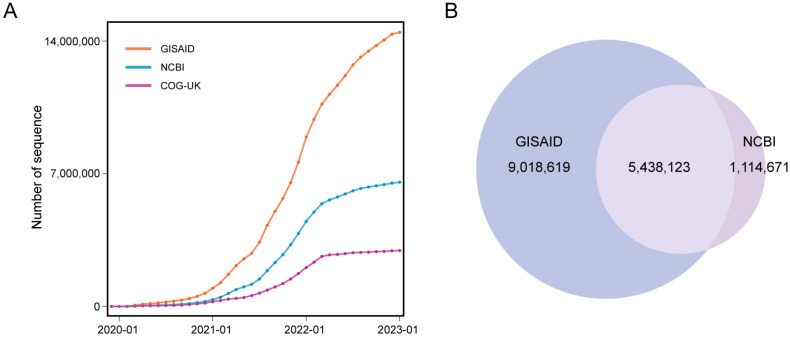
SARS-CoV-2 consensus sequences in databases. (**A**) Number of SARS-CoV-2 consensus sequences in GISAID, NCBI, and COG-UK. (**B**) Overlap of SARS-CoV-2 consensus sequences between GISAID and NCBI. This overlapping data were obtained from Nextstrain (https://data.nextstrain.org/files/ncov/open/metadata.tsv.gz, accessed on 20 February 2023).

Timely sharing of SARS-CoV-2 sequence data in public databases is important for virus surveillance and research [100]. Several databases stored and managed sequence data during the pandemic (Figure 2A). The Global Initiative on Sharing All Influenza Data (GISAID) [4,5,6] is a global data science initiative. It was launched to promote the rapid sharing of epidemic and pandemic virus data. During the COVID-19 pandemic, GISAID was one of the primary sources for consensus sequence data for SARS-CoV-2. As of January 2023, GISAID had stored more than 14 million sequences. To facilitate the sharing, access to GISAID data requires users to register and acknowledge all data contributors. In addition, there are some restrictions on GISAID data, such as the restriction on redistribution of GISAID data to any third party and the restriction on displaying GISAID data on any website without written permission. However, several researchers raised concerns about the credibility of GISAID, and they urged GISAID to acknowledge when the platform collects data from public data sets and to clearly identify those sequences [101]. The National Center for Biotechnology Information (NCBI) is one of the members of the International Nucleotide Sequence Database Collaboration (INSDC). It is a comprehensive public database that contains genomic data for various species. In light of the COVID-19 pandemic, NCBI, along with the other two members of INSDC, European Molecular Biology Laboratory-European Bioinformatics Institute (EMBL-EBI) and DNA Data Bank of Japan (DDBJ), host the same sets of SARS-CoV-2 consensus sequence data and raw sequencing data [7]. As of January 2023, NCBI had stored more than 6 million consensus sequences. The COVID-19 Genomics UK (COG-UK) Consortium [8] is a partnership of public health agencies and academic institutions in the United Kingdom. It maintains a SARS-CoV-2 genomic database to support the response to the COVID-19 pandemic. As of January 2023, COG-UK had stored more than 2 million sequences. The China National Center for Bioinformation (CNCB) RCoV19 [9,10] is a platform that collects and curates SARS-CoV-2 sequence data. It integrates sequences from GISAID, NCBI, China National GeneBank DataBase (CNGBdb), National Microbiology Data Center (NMDC), and Genome Warehouse (GWH). It is worth noting that the data in the above databases are not mutually exclusive. Some sequences were uploaded to multiple databases at the same time. For example, among the 14 million sequences in GISAID and the 6 million sequences in NCBI, more than 5 million sequences are shared by these two databases (Figure 2B). CNCB RCoV19 removes redundant sequences submitted to multiple databases and provides cross-references of such sequences for the convenience of users.

To facilitate real-time analysis and maximize the utility of openly shared data, some organizations collate the SARS-CoV-2 sequence data from public databases but do not accept direct data submissions from researchers or institutions. Nextstrain [45] collates and shares the sequence data from NCBI (https://docs.nextstrain.org/projects/ncov/en/latest/reference/remote_inputs.html, accessed on 20 February 2023). UShER [84] team provides public sequences aggregated from NCBI, COG-UK, and CNCB RCoV19 (http://hgdownload.soe.ucsc.edu/goldenPath/wuhCor1/UShER_SARS-CoV-2/, accessed on 20 February 2023) [88].

## 3. SARS-CoV-2 Genomic Annotation Web Resources

**Figure 3:**
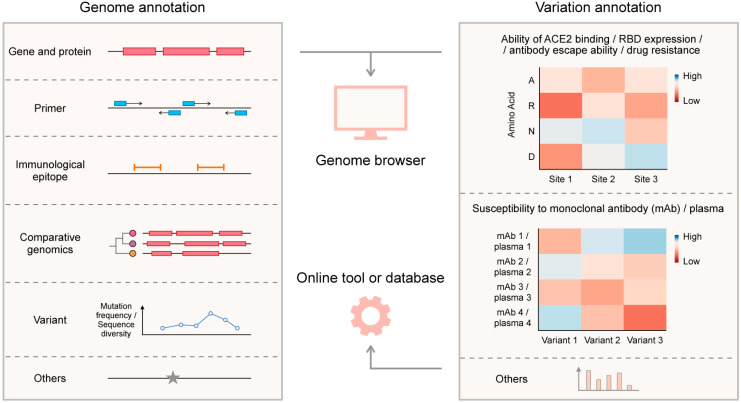
SARS-CoV-2 annotation and web resources for SARS-CoV-2 annotation. The SARS-CoV-2 annotation can be divided into genome annotation and variation annotation. Genome annotation refers to the annotation of the composition, function, and structure of the SARS-CoV-2 genome, including gene and protein, primer binding region, immunological epitope, comparison with other viruses, variant information, and other aspects. Variation annotation refers to the contribution of a single mutation or combination of multiple mutations to changes in viral properties, such as ACE2 binding, RBD expression, antibody escape ability, drug resistance, and plasma susceptibility. Genome browsers aggregate, analyze, and visualize genome annotation and variation annotation data. Some online tools or databases were designed specifically for variation annotation.

After the genome sequence of an emerging virus has been sequenced, its genome annotation is necessary. Genome annotation identifies and labels functional elements within the sequence, such as genes and proteins, primer binding regions, immunological epitopes, variation data, comparative information with other viruses, and other aspects (Figure 3). It comprehensively describes the genetic information encoded within a genome and helps researchers understand the molecular mechanisms, origin, and evolution of the virus. Many bioinformatics tools can be used for SARS-CoV-2 genome annotation [70,72]. In addition to the genome annotation of SARS-CoV-2, its variation annotation is also important. Numerous mutations have arisen in the SARS-CoV-2 genome during the pandemic, and some of them can change the transmissibility, immune escape ability, drug resistance, and other properties of the virus. Variation annotation identifies the effect of a single mutation or a combination of mutations in the virus (Figure 3). It is essential to understand the evolution and epidemiology of SARS-CoV-2 and the development of drugs and vaccines. For example, the entry receptor for SARS-CoV-2 is the angiotensin-converting enzyme 2 (ACE2), and the receptor binding domain (RBD) of the SARS-CoV-2 spike protein binds ACE2 with high affinity [102]. The RBD is also a dominant target for neutralizing antibodies [103,104,105]. Combined with real-world SARS-CoV-2 variation data, experimental measurements of how mutations in RBD affect its ACE2 binding affinity or antibody binding affinity could reveal the molecular mechanism of SARS-CoV-2 evolution [76,77,78,106,107].

Several SARS-CoV-2 genome browsers have been developed to facilitate SARS-CoV-2 genome annotation and variation annotation, including the UCSC SARS-CoV-2 Genome Browser [73], WashU SARS-CoV-2 Genome Browser [74], and Ensembl COVID-19 Browser [75] (Figure 3). These genome browsers provide interactive visualizations of the SARS-CoV-2 gene and protein annotation. In addition, with the continuous research efforts on SARS-CoV-2, its variation distribution and annotation, related virus genome comparison, diagnostic primer, and immune epitope have been investigated and reported. As these data became available to the public, they were integrated into these genome browsers and displayed in an annotation track format. In addition to these genome browsers, NCBI [7] and CNCB RCoV19 [9,10] also provide gene and protein annotations for SARS-CoV-2.

Several web tools or databases are designed specifically for SARS-CoV-2 variation annotation (Figure 3). SARS-CoV-2 RBD mutations have appeared frequently during the pandemic. Deep mutational scanning of SARS-CoV-2 RBD revealed the impact of single amino acid mutations on ACE2 binding affinity [76,77,78]. SARS-CoV-2 RBD DMS [76,77,78] interactively visualizes the deep mutational scanning data. SARS-CoV-2 RBD DMS contains two tools to help with data visualization: a set of heatmaps that display the change in ACE2 binding affinity and the change in RBD expression caused by mutations in RBD, and a plot that shows the epistatic shifts in mutational effects on ACE2 binding affinity between RBDs of different variants. To date, it contains the deep mutational scanning data for the SARS-CoV-2 wild-type, Alpha, Beta, Delta, Eta, Omicron BA.1, and Omicron BA.2 variants. Deep mutational scanning can also measure how mutations in the SARS-CoV-2 RBD affect binding by antibodies [78,106,107]. Antibody-escape estimator [79] is an interactive web resource that aggregates deep mutational scanning data from various studies to estimate the antigenic effect of mutations on RBD. It calculates and visualizes the antibody binding remaining after mutation. The type or range of antibodies can be selected by eliciting variants that they neutralize. Based on the antibody-escape estimator, it is possible to infer the next mutation steps of SARS-CoV-2 evolution to evade neutralizing antibodies. Mutation analyzer [80,81] also provides the binding affinity changes for the complexes of SARS-CoV-2 RBD and ACE2 or antibodies caused by a single mutation. CoV-RDB [82] aggregates and curates published data on the neutralizing susceptibility of SARS-CoV-2 variants and spike mutations to monoclonal antibodies, convalescent plasma, and vaccinee plasma. In addition, CoV-RDB contains another six features: (1) data aggregation for SARS-CoV-2 3C-like protease (3CLpro) inhibitor resistance mutations and RNA-dependent RNA polymerase (RdRp) inhibitor resistance mutations. (2) SARS-CoV-2 in vivo and in vitro selection data. These selection data were collected from published research. In vivo selection data contain the SARS-CoV-2 evolution within immunocompetent individuals, immunocompromised individuals, and animal hosts. CoV-RDB shows the patient’s age, immune status, infection variant, infection date, antibody treatment, and emerging spike mutations for each infection if data were available. In vitro selection data were aggregated from experiments. (3) SARS-CoV-2 variant report. For each variant of interest, CoV-RDB provides a brief description, mutation map, mutation annotation, and susceptibility summaries. (4) Mutation annotation viewers of spike, 3CLpro, and RdRp. (5) Query interface to search the website using one or more criteria: reference, monoclonal antibody, convalescent plasma, vaccine plasma, variant, and mutation. (6) A sequence analysis program that generates mutation maps, mutation annotations, and susceptibility summaries for query mutation or mutations of query sequences. CoV-RDB is a comprehensive web resource facilitating research on SARS-CoV-2 evolution, immunology, and drug development. VarEPS [83] assesses the antibody affinity, ACE2 binding affinity, and risk of amino acid substitution of SARS-CoV-2 mutations based on computational methods. It also includes an analysis program for viral sequence risk evaluation by modeling these characteristic quantities. VarEPS applies the evaluation system to sequences from public databases and generates a prewarning report based on virus growth advantage and variation risk. In addition to the annotation section, VarEPS provides a variant tracking section to analyze and display the spatiotemporal distribution and statistics for SARS-CoV-2 variation and a primer evaluation section to assess how mutations affect primers.

## 4. SARS-CoV-2 Genomic Analysis Web Tools

**Figure 4:**
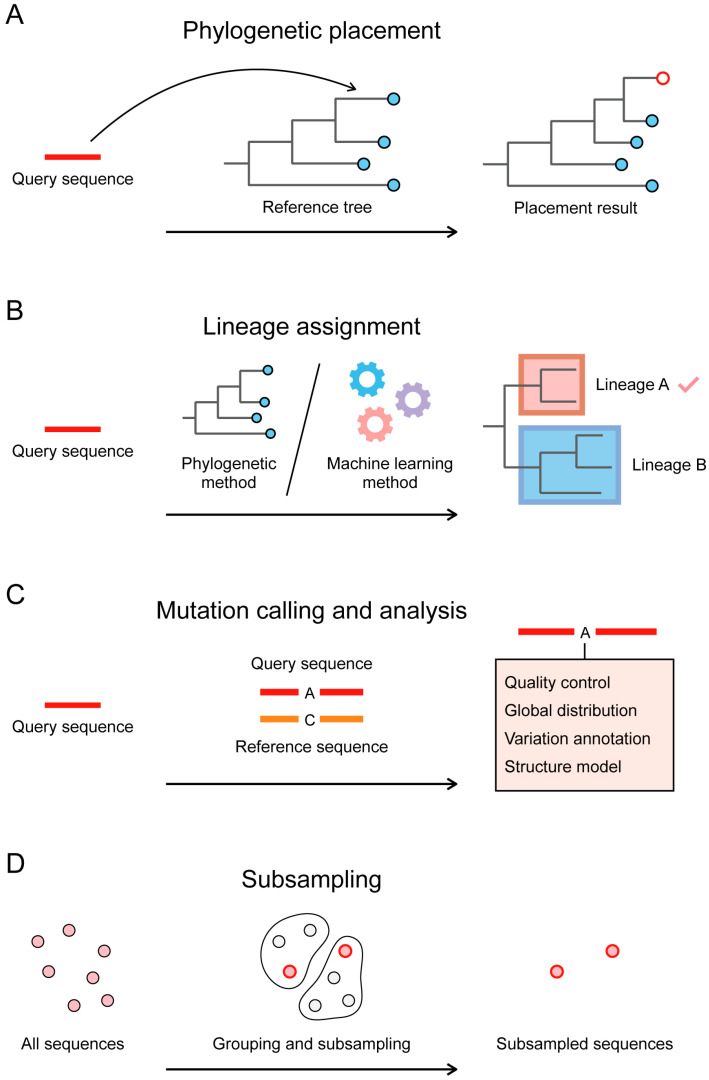
Schematic diagram of the four categories of web tools for SARS-CoV-2 genomic analysis. (**A**) Phylogenetic placement. (**B**) Lineage assignment. (**C**) Mutation calling and analysis. (**D**) Subsampling.

Due to decreasing sequencing costs and improved genomic surveillance systems, SARS-CoV-2 genomic data have reached an unprecedented number. Phylogenetic and genomic analysis of SARS-CoV-2 sequences has enabled researchers to closely track SARS-CoV-2 evolution and transmission dynamics and explore the genetic diversity of the virus. However, such massive data poses computational challenges for data analysis [88,108,109,110]. Applying existing tools for constructing, manipulating, and analyzing phylogenetic trees of large-scale SARS-CoV-2 sequences is difficult. Integrating and comparing local sequences and context sequences in public databases is also time-consuming. Many online tools were developed in this context. These tools involve the following aspects to facilitate the genomic analysis of SARS-CoV-2: phylogenetic placement, lineage assignment, mutation calling and analysis, and subsampling (Figure 4).

Currently, tens of millions of SARS-CoV-2 sequences are shared through public databases. The de novo construction of a global phylogenetic tree with so many sequences is computationally extremely difficult. Phylogenetic placement is a method for inferring a new phylogenetic tree by adding new sequences to the existing phylogenetic tree, which could reduce the use of computational resources (Figure 4A). UShER [84] is a program for rapid maximum parsimony-based placement of sequences in existing phylogenetic trees. For the query sequence, UShER computes the parsimony score considering the mutation path from the root to each node in the tree, and then places the query sequence at the node with the smallest parsimony score. The SARS-CoV-2 web application of UShER allows users to place sequences on a regularly updated global SARS-CoV-2 tree. The global tree was updated by continuously adding new sequences from public databases to the existing tree using UShER, with a starting tree derived from sarscov2phylo (https://github.com/roblanf/sarscov2phylo, accessed on 14 January 2023). After placement, UShER generates the subtree showing the query sequence in the context of its most closely related sequences.

Lineage assignment to the consensus sequence is one of the key steps for SARS-CoV-2 genomic analysis, which can reveal the genetic information from the genome sequence to help track the transmission of the virus (Figure 4B). The Pango nomenclature is a widely used lineage classification system for SARS-CoV-2 [44]. Lineages defined by this nomenclature system are known as Pango lineages. Pangolin [85] is a computational tool for assigning the most likely Pango lineage to a given SARS-CoV-2 sequence. The lineage assignment by Pangolin is based on continuously updated manual lineage designations of global sequences. These manually designated lineages and sequences are used as input for the training of pangoLEARN, an analysis mode of Pangolin. After training, pangoLEARN can be used to assign lineage to query sequences. Another analysis mode of Pangolin is UShER mode, which places query sequences on the tree with designated sequences and then infers the most likely lineage based on the placement. The UShER mode is more accurate but slower than the pangoLEARN mode [111].

Easy-to-use, fast, effective, and comprehensive mutation calling and analysis tools are needed to match the rapid sequencing of viruses (Figure 4C). CoVsurver [6] is a SARS-CoV-2 mutation calling and analysis web tool. CoVsurver maintains a database that stores published information on mutations that affect antigenic change, drug resistance, receptor binding ability, and virulence. For each query sequence, CoVsurver detects mutations in its genome and provides the global distribution information and functional annotation for each mutation. It also shows the mutations in structural models and highlights mutations close to the drug, host receptor, or antibody binding sites. Nextclade [86] is a tool for SARS-CoV-2 sequence mutation calling, quality control, lineage assignment, and phylogenetic placement. The phylogenetic placement of Nextclade is different from that of UShER. Nextclade places query sequences on a reference phylogenetic tree. It computes a distance metric, which indicates mutation difference, for the query sequence and each node in the reference tree, and then adds the query sequence near the node with the lowest distance metric. The lineage of the query sequence is assigned as the lineage of its nearest reference node during phylogenetic placement. In addition to the Pango lineage nomenclature, Nextclade also includes in its system the Nextstrain clade nomenclature, another widely used SARS-CoV-2 nomenclature.

Subsampling is another way to deal with the large data set of SARS-CoV-2 (Figure 4D). Several subsampling strategies or tools specific to SARS-CoV-2 have been developed [15,45,112] (https://github.com/nodrogluap/nybbler, accessed on 14 January 2023). covSampler [87] is a web application for subsampling SARS-CoV-2 sequences from NCBI. First, covSampler clusters sequences based on their geographic location, collection time, and genetic similarity. Then, sequences from different clusters are selected as subsamples. covSampler provides two subsampling strategies, comprehensive subsampling and representative subsampling. Comprehensive subsampling subsamples sequences from as many clusters as possible, aiming to capture a picture of the full circulating viral diversity. Representative subsampling subsamples sequences proportionally from each cluster, aiming to capture a scaled-down version of the viral population.

## 5. SARS-CoV-2 Variant Tracking Web Resources

Numerous SARS-CoV-2 variants have emerged over the course of the pandemic. Some variants may have enhanced transmissibility, immune escape ability, and virulence. Tracking the spread and outbreak of the variants in real time can facilitate researchers, policymakers, and the public to adjust control policies and public health response. However, the numerous and complex genomes and related metadata of SARS-CoV-2 pose challenges to the real-time tracking of its variants. Online web tools and dashboards that analyze, interpret, and visualize SARS-CoV-2 genomic data provide an easy way to explore the virus evolution and transmission.

Online phylogenetic trees of SARS-CoV-2 enable a better understanding and utilization of the sequence information. As mentioned above, the UShER team maintains the global phylogenetic tree by adding sequences to the existing phylogenetic tree [88]. Cov2Tree is a website for visualizing and exploring this global tree using a tool called Taxomium [89]. This tree can be zoomed in on the vertical and horizontal axes and converted between divergence-scaled and time-scaled. Cov2Tree allows users to search or color sequences according to their attributes, such as Pango lineage, geographic location, and mutation. Cluster-Tracker [90] is another web resource using the global phylogenetic tree maintained by the UShER team. It identifies and displays the United States’ SARS-CoV-2 regional introductions and transmission clusters. The algorithm of Cluster-Tracker employs a confidence metric that considers the number and distance of descendants of an internal node in a phylogenetic tree to infer whether the internal node is inside or outside a given region. The web interface of Cluster-Tracker displays the sizes, date ranges, phylogenetic lineages, and inferred origins of virus clusters in the United States. CoVizu [91] is a web platform for visualizing the global diversity and evolutionary relationships of SARS-CoV-2. CoVizu consists of two visualizations: a time-scaled phylogenetic tree of all SARS-CoV-2 Pango lineages, and a beadplot for each Pango lineage showing spatiotemporal information and evolutionary relationships of sequences within the lineage. CoVizu selects a single representative sequence for each Pango lineage to construct the phylogenetic tree of all Pango lineages. The beadplot is a custom visualization converted from phylogenetic tree. For each Pango lineage, a phylogenetic tree is constructed from sequences within the lineage using the neighbor-joining method and converted to a beadplot. Nextstrain [45] is a project to explore pathogen genome data, including surveillance views and many bioinformatics tools. For the SARS-CoV-2 surveillance view, Nextstrain subsamples thousands of sequences from global data, performs phylogenetic analysis, and displays the results in an interactive web interface. The web interface includes a phylogenetic tree, a geographic distribution map, a genome diversity view showing mutation entropy, a view of clade frequencies over time, and comprehensive search, coloring, and manipulation options. These results can be seen as a snapshot of the ongoing pandemic.

Many online dashboards for global or regional SARS-CoV-2 genomic data have been developed during the pandemic (Table 2). These dashboards continually gather, analyze, and visualize SARS-CoV-2 genomic data from different sources. They provide a convenient way to access SASR-CoV-2 genomic data by providing figures or tables and allow users to track mutation, phylogenetic lineage, geographic location, and temporal distribution of the virus. These dashboards help scientists and non-professionals with varying bioinformatics expertise tracking the virus in real time.

## 6. Discussion

The availability of web resources related to SARS-CoV-2 genomics has increased as a result of the COVID-19 pandemic. These resources help researchers understand the virus and facilitate public health responses. The databases and genomic analysis web tools facilitate global analysis of this virus. The transmission pattern and growth advantage of a new variant can be easily monitored at its early stage using variant tracking web resources combined with epidemiological information. This can serve as an early warning system to minimize the impact of any potential pandemic caused by the variant. Future growth advantage of a variant can be predicted through simulations based on data from these variant tracking web resources. It is also possible to predict the risk of a variant that has not yet emerged using annotation web resources. This prediction can be based on the experimentally measured or computationally modeled properties of the variant, including its ACE2 binding ability, potential to escape antibodies, risk of substitution, and other relevant viral characteristics. In addition, these annotation web resources benefit the field of vaccine development. Based on the risk assessment of existing and future variants, it is possible to predict the next circulating variant, which facilitates vaccine strain recommendation. The annotation web resources for antibody escape evaluation provide insights into how the virus is evolving to evade the immune system, guiding vaccine design and development.

It is worth noting that we divided these web resources into four categories (database, annotation, genomic analysis, and variant tracking) based on their main functions, while some web resources have multiple functions belonging to more than one category. GISAID [4,5,6] is not only a database, but also provides a variety of widely-used genomic dashboards and analysis tools. Similarly, both NCBI [7] and CNCB RCoV19 [9,10] store genomic sequences, feature genomic annotation views, and provide dashboard visualizations and variation overviews. CoV-RDB [82] aggregates the variation annotation data and provides a sequence analysis tool. VarEPS [83] can be used not only as a variation annotation web resource, but also to track virus transmission and analyze user-uploaded sequences.

Managing large-scale SARS-CoV-2 genomic data is a challenge for developing and maintaining SARS-CoV-2 genomic web resources. This requires efficient storage and processing systems. Many web resources have effectively accommodated such a large quantity of data. For example, CoVizu [91] and UShER [84] use the neighbor-joining method and the maximum parsimony methods instead of the more computationally intensive maximum likelihood method to construct a phylogenetic tree. CoVizu has also used asynchronous, promise-based transactions (Node.js) to reduce page load time (https://github.com/PoonLab/covizu/releases/tag/v2.0rc1, accessed on 17 January 2023). Taxonium [89], the web tool that enables Cov2Tree, uses WebGL to display web graphics using GPU and applies pruned version of trees for efficient visualization and exploration of phylogenetic trees with millions of sequences. The algorithmic improvements and reimplementation of the core algorithm from C++ to Rust have improved performance in Nextclade v2.0, compared to Nextclade v1.0 [86] (https://github.com/nextstrain/nextclade/releases/tag/2.0.0, accessed on 17 January 2023). Pangolin [85] has been optimized to increase its computation speed (https://github.com/cov-lineages/pangolin/releases/tag/v4.2, accessed on 17 January 2023). Many other web resources have also made efforts to analyze or visualize the overwhelming genomic data of SARS-CoV-2. Another effective method for dealing with the big genomic data of SARS-CoV-2 is subsampling, and we hope that more easy-to-use and reasonable subsampling algorithms or tools can be developed.

Another challenge in using, developing, and maintaining these web resources is the original data’s need for more quality and integrity. Currently, several SARS-CoV-2 genome databases collate and curate the sequence data and metadata of SARS-CoV-2. However, a downstream inspection of these data is still required. First, the sequence data should be inspected. Artifacts in the sequence may be caused by mutations incompatible with the sequencing protocol. For example, multiplex polymerase chain reaction (PCR) uses primers to attach to the viral sequence. However, mutations near the region where the primer binds may result in a reduced binding ability of the primer, causing amplicon dropout (https://community.artic.network/t/sars-cov-2-version-4-scheme-release/312, https://community.artic.network/t/sars-cov-2-v4-1-update-for-omicron-variant/342, https://community.artic.network/t/sars-cov-2-version-5-3-2-scheme-release/462, accessed on 19 January 2023). Other experimental conditions can also affect sequencing quality, such as the PCR temperature (https://community.artic.network/t/dropout-of-amplicon-64/167, accessed on 19 January 2023). Updates and developments in sequencing protocols and masking problematic sites during data processing can reduce the impact of low sequence quality in subsequent analyses. Researchers have proposed strategies for masking problematic sites of SARS-CoV-2 [113]. In addition, contamination during sequencing can result in a sequence with low quality or artifacts of co-infection or recombination. Second, the metadata of the genomic sequence should be inspected. Inaccurate or incomplete metadata (including but not limited to: collection time, location, sequencing method, bioinformatics analysis method, sequencing and uploading laboratory, and host information) of virus sequence may cause obstacles or misinterpretations. The sampling and sequencing bias should also be considered when analyzing and interpreting the SARS-CoV-2 sequence data.

Maintenance of the web resources helps to optimize the performance and to keep the content relevant and accurate. As mentioned above, some web resources have updated their algorithms or adopted new methods to improve their performance. New features can also be added to improve the user experience. In addition, it is necessary to update with new data regularly for web resources using the increasing genomic data or aggregating new results. Due to limited funding or other constraints, some web resources for SARS-CoV-2 genomics are no longer updated with new data, and their results are of limited significance. These web resources are not included in this review. Version control of web resources is also important during maintaining or updating. It allows developers to track changes and easily identify and fix issues. In addition, reproducibility is an important aspect of scientific research and data analysis. Version control also helps users to reproduce and validate the results obtained from the web resources.

Coordination and interaction between these web resources improve the efficiency of analysis of viral evolution and spread. GISAID [4,5,6] links to the Nextstrain platform [45], Outbreak.info [93,94], and CoVizu [91] to show the global and regional spread and evolution of SARS-CoV-2. CoVsurver [6] is also embedded in GISAID, allowing users to analyze sequences deposited in the database. covSpectrum [92] can send a list of sequences to UShER [84] for analysis and to Taxonium [89] for visualization. The resulting subtree of phylogenetic placement by UShER can be visualized using Auspice (https://auspice.us, accessed on 6 February 2023), which is part of the Nextstrain project. cov-lineages.org [98] links to Outbreak.info for details on Pango lineages. These interactions provide users with more comprehensive and integrated information and experience. We encourage existing and new web resources to strengthen their connections with other resources.

The web resources about SARS-CoV-2 genomics help us understand the spread and evolution of SARS-CoV-2. These resources benefit from the generous sharing of sequencing, experimental, and computational data. These data play an important role in the global effort to control the pandemic and protect public health.

## Figures and Tables

**Table 1:** Summary of web resources for the SARS-CoV-2 genomic database, annotation, analysis, and variant tracking.

Web resource	Link	Reference
**Database**		
GISAID	https://gisaid.org/, accessed on 20 February 2023	[4,5,6]
NCBI	https://www.ncbi.nlm.nih.gov/sars-cov-2/, accessed on 20 February 2023	[7]
COG-UK	https://www.cogconsortium.uk/priority-areas/data-linkage-analysis/public-data-analysis/, accessed on 20 February 2023	[8]
CNCB RCoV19	https://ngdc.cncb.ac.cn/ncov/release_genome, accessed on 20 February 2023	[9,10]
**Annotation**		
UCSC SARS-CoV-2 Genome Browser	https://genome.ucsc.edu/covid19.html, accessed on 12 January 2023	[73]
WashU SARS-CoV-2 Genome Browser	https://virusgateway.wustl.edu/, accessed on 12 January 2023	[74]
Ensembl COVID-19 Browser	https://covid-19.ensembl.org, accessed on 12 January 2023	[75]
NCBI SARS-CoV-2 Annotation	https://www.ncbi.nlm.nih.gov/nuccore/NC_045512.2?report=graph, accessed on 11 January 2023	[7]
CNCB RCoV19 Annotation	https://ngdc.cncb.ac.cn/ncov/knowledge/gene, accessed on 13 January 2023	[9,10]
SARS-CoV-2 RBD DMS	https://jbloomlab.github.io/SARS-CoV-2-RBD_DMS/, accessed on 13 January 2023 https://jbloomlab.github.io/SARS-CoV-2-RBD_DMS_variants/, accessed on 13 January 2023 https://jbloomlab.github.io/SARS-CoV-2-RBD_DMS_Omicron/, accessed on 13 January 2023	[76,77,78]
Antibody-escape estimator	https://jbloomlab.github.io/SARS2_RBD_Ab_escape_maps/escape-calc/, accessed on 13 January 2023	[79]
Mutation analyzer	https://weilab.math.msu.edu/MutationAnalyzer/, accessed on 13 January 2023	[80,81]
CoV-RDB	https://covdb.stanford.edu/, accessed on 14 January 2023	[82]
VarEPS	https://nmdc.cn/ncovn/, accessed on 18 January 2023	[83]
**Analysis**		
UShER	https://genome.ucsc.edu/cgi-bin/hgPhyloPlace, accessed on 14 January 2023	[84]
Pangolin	https://pangolin.cog-uk.io/, accessed on 14 January 2023	[85]
CoVsurver	https://corona.bii.a-star.edu.sg/, accessed on 14 January 2023	[6]
Nextclade	https://clades.nextstrain.org/, accessed on 14 January 2023	[86]
covSampler	https://www.covsampler.net/, accessed on 14 January 2023	[87]
**Variant tracking**		
Cov2Tree	https://cov2tree.org/, accessed on 15 January 2023	[88,89]
Cluster-Tracker	https://clustertracker.gi.ucsc.edu/, accessed on 15 January 2023	[90]
CoVizu	https://filogeneti.ca/CoVizu/, accessed on 15 January 2023	[91]
Nextstrain	https://nextstrain.org/, accessed on 15 January 2023	[45]
CoVariants	https://covariants.org/, accessed on 15 January 2023	/
covSpectrum	https://cov-spectrum.org/, accessed on 15 January 2023	[92]
Outbreak.info	https://outbreak.info/, accessed on 15 January 2023	[93,94]
COVID CG	https://covidcg.org/, accessed on 15 January 2023	[95]
CoVerage	https://sarscoverage.org/, accessed on 15 January 2023	/
CovGlobe	https://covglobe.org/, accessed on 15 January 2023	/
REGENERON COVID-19 Dashboard	https://covid19dashboard.regeneron.com/, accessed on 15 January 2023	/
COVID-19 Viral Genome Analysis Pipeline	https://cov.lanl.gov/, accessed on 15 January 2023	[96]
CovMT	https://www.cbrc.kaust.edu.sa/covmt/, accessed on 15 January 2023 https://www.cbrc.kaust.edu.sa/covmtdev/, accessed on 15 January 2023	[97]
cov-lineages.org	https://cov-lineages.org/, accessed on 15 January 2023	[98]
SARS-CoV-2 Africa dashboard	https://climade.health/dashboard/covid-africa/, accessed on 15 January 2023	[99]
Wellcome Sanger Institute COVID-19 Genomic surveillance dashboard	https://covid19.sanger.ac.uk/, accessed on 15 January 2023	/
covidtag	http://covidtag.paseq.org/, accessed on 15 January 2023	/

**Table 2:** Summary of SARS-CoV-2 genomic online dashboards.

Web Resource	Data Source	Region
CoVariants	GISAID	Global
covSpectrum	GISIAD and NCBI	Global
Outbreak.info	GISAID	Global
COVID CG	GISAID	Global
CoVerage	GISAID	Global
CovGlobe	GISAID	Global
Regeneron COVID-19 Dashboard	GISAID	Global
COVID-19 Viral Genome Analysis Pipeline	GISAID	Global
CovMT	GISAID	Global
cov-lineages.org	GISAID	Global
SARS-CoV-2 Africa Dashboard	GISAID	Africa
Wellcome Sanger Institute COVID-19 Genomic Surveillance Dashboard	COG-UK	England
covidtag	GISAID	Spain

## Data Availability

Not applicable.
